# The relationship between follicle-stimulating hormone and metabolic dysfunction-associated fatty liver disease in men

**DOI:** 10.1038/s41387-024-00314-1

**Published:** 2024-07-11

**Authors:** Dong-Hua Bin, Fang Liu, Ke-Ping Peng, Min Zhan, Yan Tan, Qiao Liu, Wang Tang, Zeng-Nan Mo, Xiong-Jun Peng, Gui-Xiang Tian

**Affiliations:** 1 Department of Anus and Intesine, The First Hospital of Hunan University of Chinese Medicine, Changsha, China; 2 Department of Ultrasoud, The First Hospital of Hunan University of Chinese Medicine, Changsha, China; 3 Department of Otorhinolaryngology-Head and Neck surgery, The first Hospital, Hunan University of Chinese Medicine, Changsha, China; 4 Department of Ultrasoud, The Second Xiangya Hospital, Central South University, Changsha, China; 5 Centre for Genomic and Personalized Medicine, Guangxi Medical University, Nanning, China; 6 Department of Medical Equipment, The Second Xiangya Hospital, Central South University, Changsha, China

**Keywords:** Risk factors, Epidemiology

## Abstract

**Objectives:**

The present study aimed to investigate the relationship between male hormones and metabolic dysfunction-associated fatty liver disease (MAFLD) in males.

**Methods:**

Data from the Fangchenggang Area Male Health and Examination Survey (FAMHES) were used to analyze the male hormone levels between MAFLD patients and controls. Univariate and multivariate logistic regression analyses were performed to identify risk factors for MAFLD. Receiver operating characteristic curve analysis was used to assess the diagnostic performance of male hormones for MAFLD.

**Result:**

A total of 1578 individuals were included, with 482 individuals (30.54%) of MAFLD, including 293 (18.57%) with mild disease and 189 (11.98%) with moderate-to-severe disease. The MAFLD patients were significantly older than those without MAFLD. The LH, FSH, and SHBG levels in the MAFLD patients were significantly greater than those in the control group. Age, FSH, LH, SHBG, and estradiol were all risk factors for MAFLD. Age, FSH, and LH were risk factors for moderate-to-severe MAFLD. FSH was an independent risk factor for MAFLD and moderate-to-severe MAFLD. FSH showed an excellent diagnostic value, with an AUC of 0.992 alone and 0.996 after adjusting age.

**Conclusions:**

Our findings indicate that FSH may be a potential diagnostic and predictive biomarker for MAFLD.

## Introduction

Metabolic dysfunction-associated fatty liver disease (MAFLD), formerly known as nonalcoholic fatty liver disease (NAFLD), is the most common cause of chronic liver disease worldwide and can progress to liver fibrosis, cirrhosis, and even hepatocellular carcinoma [1–4]. Therefore, MAFLD is a major public health issue. A meta-analysis conducted in 2016 revealed that the prevalence of MAFLD in the general population is 25.24%, with the highest prevalence in the Middle East (31.79%) and South America (30.45%), and the lowest in Africa (13.48%) [5]. Another meta-analysis conducted in 2019 reported that the pooled prevalence of MAFLD in China is 29.2%, and it has been increasing rapidly, from 25.4% in 2008–2010 to 32.3% in 2015–2018 [6]. With the rising incidence of obesity worldwide, the prevalence of MAFLD is also growing rapidly [3, 7]. Currently, a liver biopsy is regarded as the gold standard for clinically diagnosing and staging MAFLD [7, 8]. However, its invasive nature makes it challenging to perform in routine clinical practice. Therefore, the identification of novel biomarkers associated with MAFLD has become crucial for the early detection and assessment of MAFLD severity [9].

MAFLD is clinically highly associated with metabolic syndrome [10]. Besides causing significant pathological changes in the liver, MAFLD also affects the function of other organs and systems, especially the endocrine system [11, 12]. MAFLD is a sex-dimorphic disease, with a generally greater prevalence in men [13, 14]. Gender and sex hormones have a significant impact on various factors associated with MAFLD, such as genetic variants, cytokines, stress, and environmental factors, which can modify the risk profiles and phenotypes of MAFLD in individuals [14, 15].

Testosterone is the major male hormone and is responsible for regulating many metabolic processes, such as fat metabolism, insulin sensitization, and suppression of lipogenesis, besides maintaining normal sexual function and reproductive ability [16]. Several studies have investigated the relationship between testosterone levels and MAFLD, but the results are inconsistent. Some studies have reported that a lower total testosterone (TT) level is associated with a high prevalence of MAFLD and adverse clinical outcomes [17–19], while other studies have shown that there is no significant association between the testosterone level and MAFLD [20, 21]. Luteinizing hormone (LH) and sex hormone-binding globulin (SHBG) are two key regulatory factors that regulate testosterone levels [22–24]. LH can promote testosterone secretion by Leydig cells in the testis [24], while SHBG can bind to testosterone and affect its bioavailability in the body [22, 23]. A recent meta-analysis has shown that although a lower testosterone level is associated with the severity of MAFLD, the relationship between SHBG and the severity of MAFLD remains controversial [22]. For example, a higher SHBG level was associated with the severity of MAFLD in men with a body mass index >27 kg/m^2^, while a lower SHBG level was associated with the severity of MAFLD in men older than 50 years old [22]. Two other studies found that SHBG was negatively correlated with MAFLD in men [25, 26]. Cao and collaborators showed that there was no association between LH and MAFLD in men, whereas women with MAFLD had significantly lower levels of LH than those without MAFLD [25]. Furthermore, follicle-stimulating hormone (FSH) is an important hormone regulating the proliferation and maturation of germ cells [24]. FSH is reported to be associated with MAFLD in postmenopausal women [27–29]; however, studies regarding the relationship between FSH and MAFLD in men are limited.

Therefore, the present study aimed to investigate the relationship between male hormones (LH, SHBG, FSH, and testosterone) and MAFLD in males, hoping to provide novel biomarkers for the early identification of MAFLD severity and prognosis in patients with MAFLD.

## Materials and methods

### Participants

This study was conducted using the data from the Fangchenggang Area Male Health and Examination Survey (FAMHES), which is a population-based survey conducted from September 2009 to December 2009 among noninstitutionalized Chinese males aged 17–88 years old in Guangxi, China, and has been used in many previous studies [30–34]. The aim of FAMHES was to investigate the impact of environmental and genetic factors, as well as their interactions, on the development of age-related chronic diseases. A comprehensive population and health questionnaire was provided to 4303 male participants at the Medical Center of the First People’s Hospital of Fangchenggang, and extensive physical examinations were conducted. Written informed consent was obtained from all participants. This human data is in accordance with the Declaration of Helsinki. and the study was approved by the Guangxi ethics committee.

All participants were requested for follow-up, which included another health questionnaire and biochemical analysis of a fasting blood sample collected in the morning. A trained expert measured the height and weight of the participants using standard procedures.

Participants were excluded from this study if any of the following conditions were met: (I) currently diagnosed with diabetes, coronary heart disease, stroke, hyperthyroidism, rheumatoid arthritis, inflammatory disease of the reproductive system, urinary diseases (infection, stone, hematuria, or hematospermia), prostatitis or history of any cancers; (II) taking any medication; (III) with impaired liver function (alanine aminotransferase >2.0 times the upper limit of normal); (IV) with a history of excessive alcohol consumption (>40 mL/d); or (V) with impaired kidney function (serum creatinine >178 mmol/L).

**Fig. 1:**
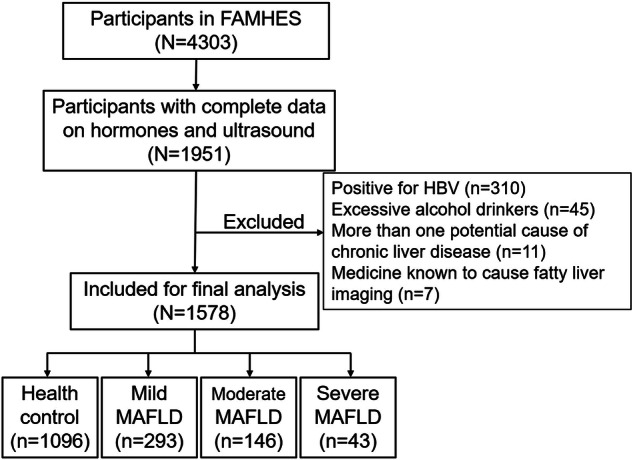
Schematic diagram to show the enrollment of the participants. FAMHES Fangchenggang Area Male Health and Examination Survey, HBV hepatitis B virus, MAFLD metabolic dysfunction-associated fatty liver disease.

After excluding participants who did not undergo complete clinical and laboratory tests or ultrasound examinations, a total of 1951 participants were available for analysis. It was found that 310 participants were positive for hepatitis B virus (determined by hepatitis B surface antigen detection), 45 participants were excessive alcohol drinkers (>40 mL/d), 11 participants had at least one potential cause of chronic liver disease, and seven participants were taking medications known to cause fatty liver imaging. After excluding these participants, 1578 males were finally included in this study (Fig. 1).

### Ultrasound examination

Two experienced ultrasound doctors [Cohen’s kappa of 0.621 (95% CI 0.352–0.876)] performed abdominal ultrasound examinations on all patients using a portable ultrasound device (GE, LOGIQ, 5.0 MHz transducer, USA). The size, contour, echogenicity, structure, and posterior attenuation of the liver were assessed for each participant. The ultrasound criteria for diagnosing a fatty liver included increased liver echogenicity (bright), liver parenchymal echogenicity greater than that of the renal parenchyma, and blurring and narrowing of the hepatic vein lumen [35, 36]. According to the criteria described by Saadeh et al. [36], all participants were divided into two groups (mild and moderate-to-severe).

### Laboratory tests

In the laboratory tests of FAMHES, all participants were required to fast overnight. Samples containing approximately 10 mL of venous blood were collected from 8 am to 11 am the next day, frozen (~2 h), and sent to the testing center of the First Affiliated Hospital of Guangxi Medical University in Nanning. The samples were centrifuged within 15–25 min and then stored at −80 °C until analysis. The serum samples were thawed at room temperature for 1 h and then analyzed by inverting the test tube ten times. All analyses were performed by the same operator using the same batch of reagents.

The blood lipid parameters were measured on an automated analyzer (Dade Behring, USA) in the laboratory at the Fangchenggang. The FSH, LH, SHBG, estradiol (E2), and testosterone levels were determined using the COBAS 6000 system E601 (Elecsys module) and an immunoassay analyzer (Roche Diagnostics GmbH, Mannheim, Germany). The inter-assay coefficients of variation were 3.6% for E2, 4.4% for SHBG, 4.3% for FSH, and 3.6% for LH. The lower limit of detection for the assay was 0.05 pg/mL. Hormones were measured in nonfasting blood samples that were collected within 4 h after waking up, thus balancing the diurnal variation of hormone levels. All measurements were performed according to the manufacturer’s instructions.

### Statistical analysis

Statistical analysis was performed using SPSS 20.0. Data distribution was analyzed by the Kolmogorov–Smirnov and Shapiro–Wilk tests. Continuous data with a normal distribution were expressed as the mean ± standard derivation, while continuous data with a non-abnormal distribution were expressed as the median (range). Univariate logistic regression analysis was performed to identify risk factors for MAFLD or moderate-to-severe MAFLD in all collected factors. Parameters with a *P* value less than 0.1 in univariate analysis entered multivariate logistic regression analysis to identify independent risk factors for MAFLD or moderate-to-severe MAFLD. The variance inflation factor was calculated to analyze the multicollinearity and variables with multicollinearity were excluded from the multivariate logistic regression. The receiver operating characteristic (ROC) curve was plotted, and the area under the curve (AUC) with 95% confidence interval (CI) was used to assess the diagnostic performance of male hormones for MAFLD. A *P* value less than 0.05 was considered statistically significant.

## Results

### Baseline characteristics of the included individuals

Of the included 1578 subjects, 482 individuals (30.54%) were diagnosed with MAFLD, with 293 (18.57%) having mild disease and 189 (11.98%) having moderate-to-severe disease (Table 1). Therefore, these participants were divided into three groups: control (1096 cases), mild MAFLD (293 cases), and moderate-to-severe MAFLD (189 cases).

**Table 1:** Baseline characteristics of the study subjects with or without MAFLD.

Characteristic	Ultrasonographic liver steatosis (Total = 482)					Age-adjusted
	Control (*n* = 1096)	Mild MAFLD (*n* = 293)	Moderate-to-severe MAFLD (*n* = 189)	F	*P* value	*P* value
Age (years)	35.18 ± 9.06	41.43 ± 9.86*	46.42 ± 11.09*#	141.469	<0.001	
Height(cm)	168.19 ± 5.57	167.97 ± 5.77	167.66 ± 5.36	0.801	0.449	
Weight (kg)	66.09 ± 10.78	66.16 ± 9.96	66.28 ± 9.72	0.027	0.973	
BMI (kgm^−2^)	23.33 ± 3.43	23.43 ± 3.19	23.52 ± 2.99*	0.293	0.746	
Waist (cm)	80.53 ± 9.40	81.81 ± 9.32*	82.26 ± 9.17*#	4.178	0.015	0.046
Buttock (cm)	91.61 ± 6.54	91.49 ± 5.83	91.61 ± 5.45	0.047	0.954	
WHR	0.88 ± 0.06	0.89 ± 0.06*	0.89 ± 0.06*	12.607	<0.001	0.109
SBP (mmHg)	116.65 ± 13.88	119.25 ± 16.79*	122.85 ± 18.27*#	15.191	<0.001	0.087
DBP (mmHg)	76.59 ± 9.92	77.79 ± 11.02	78.56 ± 10.71*	3.922	0.020	0.041
ALT (mmol L^−1^)	46.77 (30.00–55.56)	47.67 (32.00–56.50)	43.41 (31.00–51.00)*#	1.767	0.171	
GLU	5.28 ± 0.98	5.40 ± 1.16	5.42 ± 0.98	2.623	0.073	
TC (mmol L^−1^)	5.67 ± 1.02	5.88 ± 1.08*	5.96 ± 1.01*	8.935	<0.001	<0.001
TGG (mmol L^−1^)	1.57 (0.77–1.80)	1.52 (0.76–1.67)	1.42 (0.76–1.74)	0.692	0.501	
HDL (mmol L^−1^)	1.39 ± 0.33	1.42 ± 0.37	1.44 ± 0.28	2.091	0.124	
LDL (mmol L^−1^)	2.95 ± 0.82	3.12 ± 0.79*	3.15 ± 0.75*	8.363	<0.001	0.037
FSH (mIU mL^−1^)	4.56 ± 1.05	7.75 ± 0.78*	10.54 ± 1.26*#	3276.718	<0.001	<0.001
LH (mIU mL^−1^)	5.02 (3.86–5.93)	6.12 (4.70–7.38)*	7.16 (5.39–8.62)*#	128.040	<0.001	<0.001
SHBG (nmol L^−1^)	39.59(26.97–49.10)	46.27 (29.91–56.47)*	49.61 (32.90–61.58)*	25.802	<0.001	0.001
TT (ng mL^−1^)	6.26 (4.94–7.41)	6.37 (5.00–7.56)	6.19 (4.86–7.33)	0.538	0.584	
E2 (pg mL^−1^)	34.64 ± 10.12	33.44 ± 9.92	31.98 ± 9.55*	6.438	0.002	0.008

*P < 0.05 compared with the control group; #*P* < 0.05 compared with the mild group.

*ALT* alanine transaminase, *BMI* body mass index, *CHOL* cholesterol, *DBP* diastolic blood pressure, *E2* estradiol, *FSH* follicle-stimulating hormone, *GLU* blood glucose, *HDL* high-density lipoprotein, *LDL* low-density lipoprotein, *LH* luteinizing hormone, *SBP* systolic blood pressure, *SHBG* sex hormone-binding globulin, *TC* total cholesterol, *TG* triglyceride, *TT* total testosterone, *WHR* waist–hip ratio.

The MAFLD patients was significantly older than those without MAFLD (*P* < 0.001), and the patients with moderate-to-severe MAFLD were older than those with mild MAFLD (*P* < 0.05). The LH, FSH, and SHBG were significantly greater in the MAFLD patients compared to the control group (*P* < 0.001). In addition, the LH, and FSH levels were greater in the patients with moderate-to-severe MAFLD compared to those with mild MAFLD (*P* < 0.05), while there was no significant difference in the SHBG levels between the patients with moderate-to-severe MAFLD and those with mild MAFLD (Table 1). Moreover, the levels of LH, FSH, and SHBG were all significantly greater in the MAFLD patients than in the control group among different age groups and increased in an age-dependent manner (*P* < 0.05, Table 2). Since age was a confounder for MAFLD, the parameters with a *p* value less than 0.05 in Table 1 was further analyzed using age as a covariate. After adjusting age, LH, FSH, and SHBG remained significantly higher in the MAFLD patients compared to the control group (Table 1).

**Table 2:** Baseline characteristics of the study subjects.

Characteristic	Age						Group	Group*Age
	<35years old (*n* = 669)		35–45 years old (*n* = 585)		>45 years old (*n* = 324)			
	Control (*n* = 566)	MAFLD (*n* = 103)	Control (*n* = 388)	MAFLD (*n* = 197)	Control (*n* = 142)	MAFLD (*n* = 182)	*P*	*P*
Height (cm)	168.60 ± 5.56	168.52 ± 5.44	168.04 ± 5.63	168.27 ± 5.80	166.55 ± 5.47	166.86 ± 5.02	0.635	0.885
Weight (kg)	64.55 ± 10.88	64.30 ± 10.13	67.90 ± 10.43	67.96 ± 10.22	67.07 ± 10.14	66.90 ± 9.06	0.847	0.937
BMI (kgm^−2^)	22.69 ± 3.45	22.62 ± 3.26	24.02 ± 3.33	23.96 ± 3.05	24.14 ± 3.20	24.01 ± 2.97	0.658	0.984
Waist (cm)	78.249 ± 9.01	78.45 ± 9.08	82.80 ± 8.86	82.78 ± 9.15	83.96 ± 8.94	83.99 ± 9.11	0.895	0.983
Buttock (cm)	90.87 ± 6.78	90.28 ± 5.69	92.38 ± 6.11	92.41 ± 5.71	92.56 ± 6.09	92.09 ± 5.37	0.345	0.734
WHR	0.86 ± 0.05	0.87 ± 0.06	0.90 ± 0.56	0.89 ± 0.59	0.91 ± 0.05	0.91 ± 0.61	0.262	0.514
SBP (mmHg)	114.72 ± 11.92	116.53 ± 13.38	117.78 ± 14.32	118.48 ± 14.26	122.21 ± 17.10	129.06 ± 20.14*	0.000	0.006
DBP (mmHg)	75.02 ± 8.43	74.96 ± 10.03	77.92 ± 10.42	78.78 ± 9.99	79.43 ± 11.62	80.79 ± 11.94	0.239	0.660
ALT (mmol L^−1^)	46.64 (28.00–56.00)	47.27 (30.00–57.00)	48.85(31.25–58.00)	47.34 (32.00–56.00)	43.50 (31.00–50.00)	44.62 (31.00–54.00)	0.940	0.717
GLU	5.14 ± 0.65	5.33 ± 1.40*	5.32 ± 0.75	5.38 ± 1.07	5.78 ± 1.66	5.56 ± 1.14	0.720	0.033
TC (mmol L^−1^)	5.49 ± 0.99	5.72 ± 1.10*	5.81 ± 0.97	5.89 ± 1.00	5.90 ± 1.02	6.11 ± 1.08	0.002	0.410
TGG (mmol L^−1^)	1.44 (0.74–1.70)	1.63 (0.69–1.72)	1.678 (0.86–1.89)	1.50 (0.83–1.82)	1.71 (0.84–2.10)	1.46 (0.75–1.68)	0.684	0.217
HDL (mmol L^−1^)	1.38 ± 0.28	1.43 ± 0.43	1.37 ± 0.32	1.43 ± 0.32*	1.36 ± 0.31	1.46 ± 0.34*	0.000	0.723
LDL (mmol L^−1^)	2.84 ± 0.82	2.96 ± 0.80	3.05 ± 0.77	3.08 ± 0.76	3.09 ± 0.86	3.28 ± 0.79*	0.007	0.272
FSH (mIU mL^−1^)	4.33 ± 1.03	8.17 ± 1.63*	4.74 ± 1.04	8.64 ± 1.61*	4.95 ± 1.03	9.24 ± 1.63*	0.000	0.071
LH (mIU mL^−1^)	5.06 (3.92–5.98)	6.38 (4.84–7.33)*	4.83 (3.65–5.83)	6.46 (4.77–7.76)*	4.92 (4.09-5.97)	6.67 (5.50-8.17)*	0.000	0.363
SHBG (nmol L^−1^)	37.45 ± 15.88	39.43 ± 19.02	39.77 ± 18.50	45.19 ± 24.97*	47.38 ± 28.79	51.22 ± 23.29*	0.002	0.454
TT (ng mL^−1^)	6.54 (5.20–7.72)	6.48 (5.09–7.84)	5.98 (4.60–7.06)	6.14 (4.92–7.09)	5.88 (4.57–6.76)	6.10 (4.71–7.20)	0.323	0.520
E2 (pg mL^−1^)	34.98 ± 9.48	31.39 ± 8.92*	34.02 ± 10.38	31.61 ± 9.51*	35.79 ± 11.74	34.14 ± 10.44	0.000	0.434

*P < 0.05 compared with the control group.

*ALT* alanine transaminase, *BMI* body mass index, *CHOL* cholesterol, *DBP* diastolic blood pressure, *E2* estradiol, *FSH* follicle-stimulating hormone, *GLU* blood glucose, *HDL* high-density lipoprotein, *LDL* low-density lipoprotein, *LH* luteinizing hormone, *SBP* systolic blood pressure, *SHBG* sex hormone-binding globulin, *TC* total cholesterol, *TG* triglyceride, *TT* total testosterone, *WHR* waist–hip ratio.

### Logistic regression analysis for the risk factors of MAFLD

Univariate logistic regression analysis showed that age, waist–hip ratio (WHR), systolic blood pressure (SBP), diastolic blood pressure (DBP), blood glucose (GLU), cholesterol (CHOL), high-density lipoprotein (HDL), low-density lipoprotein (LDL), FSH, LH, SHBG, and E2 were all risk factors for the occurrence of MAFLD (all *P* < 0.05) (Table 3). Multivariate logistic regression analysis identified that only FSH was an independent risk factor for MAFLD (*P* < 0.001) (Table 3).

**Table 3:** Logistic regression analysis for the risk factors of MAFLD.

Characteristic	Total (*N*)	Univariate analysis		Multivariate analysis	
		Odds Ratio (95% CI)	*P* value	Odds Ratio (95% CI)	*P* value
Age	1578	1.086 (1.073–1.099)	<0.001	1.027 (0.941–1.121)	0.546
Age (years)	1578				
<35	669	Reference		Reference	
35–45	585	3.159 (2.403–4.151)	<0.001	0.736 (0.196–2.765)	0.649
>45	324	7.153 (5.264–9.719)	<0.001	0.443 (0.041–4.813)	0.504
Height	1578	0.990 (0.971–1.009)	0.312		
Weight	1578	1.001 (0.991–1.011)	0.847		
Waist	1576	1.017 (1.005–1.028)	0.005	1.012(0.992-1.031)	0.237
Buttocks	1575	0.998 (0.981–1.015)	0.839		
SBP	1578	1.017 (1.010–1.024)	<0.001	1.004 (0.971–1.039)	0.807
DBP	1578	1.014 (1.004–1.025)	0.008	1.007 (0.958–1.057)	0.796
ALT	1578	0.999 (0.995–1.003)	0.580		
GLU	1578	1.120 (1.013–1.239)	0.027	1.019 (0.775–1.339)	0.894
CHOL	1562	1.241 (1.119–1.376)	<0.001	0.796 (0.299–2.122)	0.648
TG	1562	0.966 (0.900–1.037)	0.337		
HDL	1562	1.376 (1.004–1.884)	0.047	1.052 (0.220–5.032)	0.949
LDL	1562	1.315 (1.151–1.502)	<0.001	1.217 (0.355–4.169)	0.754
FSH	1578	70.673 (34.346– 145.423)	<0.001	132.284 (54.034– 323.852)	< 0.001
LH	1578	1.492 (1.402–1.588)	<0.001	1.003 (0.806–1.248)	0.978
SHBG	1578	1.018 (1.012–1.023)	<0.001	1.000 (0.951–1.051)	0.992
TT	1578	1.010 (0.956–1.068)	0.712		
E2	1574	0.982 (0.971–0.993)	0.001	0.990 (0.946–1.037)	0.674

Parameters with a *P* value less than 0.1 in univariate analysis entered multivariate logistic regression analysis.

*BMI* body mass index, *WHR* waist–hip ratio, *SBP* systolic blood pressure, *DBP* diastolic blood pressure, *ALT* alanine transaminase, *GLU* blood glucose, *CHOL* cholesterol, *TC* total cholesterol, *TG* triglyceride, *HDL* high-density lipoprotein, *LDL* low-density lipoprotein, *FSH* follicle-stimulating hormone, *LH* luteinizing hormone, *SHBG* sex hormone-binding globulin, *TT* total testosterone, *E2* estradiol.

According to the univariate regression analysis comparing the severe MAFLD patients with the mild MAFLD patients, age, FSH, and LH were found to be risk factors for severe MAFLD (all *P* < 0.05). Compared to the patients aged >45 years old, those aged <35 years old (OR = 0.341, 95% CI: 0.197–0.589) or 35–45 years old (OR = 0.598, 95% CI: 0.398–0.898) had a reduced risk of moderate-to-severe MAFLD (*P* < 0.05) (Table 4). Further multivariate regression analysis revealed that only FSH was an independent risk factor for moderate-to-severe MAFLD (*P* < 0.001) (Table 4).

**Table 4:** Logistic regression analysis for the risk factors of moderate-to-severe MAFLD.

Characteristic	Total (*N*)	Univariate analysis		Multivariate analysis	
		Odds ratio (95% CI)	*P* value	Odds ratio (95% CI)	*P* value
Age Section	482				
>45	180	Reference		Reference	
<35	96	0.341 (0.197–0.589)	<0.001	0.464 (0.130–1.660)	0.238
35–45	206	0.598 (0.398–0.898)	0.013	0.945 (0.387–2.304)	0.900
FSH	482	28.311 (13.943–57.486)	<0.001	29.157 (14.110–60.250)	<0.001
LH	482	1.249 (1.142–1.365)	<0.001	1.005 (0.822–1.229)	0.961
SHBG	482	1.005 (0.998–1.012)	0.158		
TT	482	0.959 (0.877–1.049)	0.366		
E2	480	0.985 (0.966–1.004)	0.113		

Parameters with a *P* value less than 0.1 in univariate analysis entered multivariate logistic regression analysis.

*FSH* follicle-stimulating hormone, *LH* luteinizing hormone, *SHBG* sex hormone-binding globulin, *TT* total testosterone, *E2* estradiol.

### Diagnostic value of male hormones for MAFLD

**Fig. 2:**
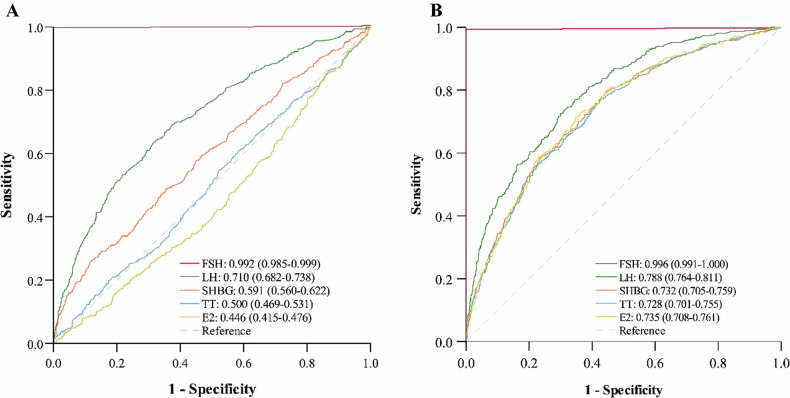
ROC curves of male hormones for MAFLD. ROC curves of male hormones without (**A**) or with age adjustment (**B**). E2 estradiol, FSH follicle-stimulating hormone, LH luteinizing hormone, SHBG sex hormone-binding globulin, TT total testosterone. AUCs are presented as AUC (95% CI).

We also analyzed the diagnostic values of LH, FSH, TT, SHBG, and E2 for MAFLD. As shown in Fig. 2A, FSH showed an excellent diagnostic value, with an AUC of 0.992 (95% CI: 0.985–0.999); LH had an AUC of 0.710 (95% CI: 0.682–0.738); while TT, SHBG, and E2 only had AUC values of 0.446–0.597. After adjusting the age confounder, AUCs of all hormones were increased, with FSH presenting an AUC of 0.996 (95% CI: 0.991–1.000) and LH presenting an AUC of 0.788 (0.764–0.811) (Fig. 2B).

## Discussion

In the present study, we found that age, WHR, SBP, DBP, GLU, CHOL, HDL, LDL, FSH, LH, SHBG, and E2 were all risk factors for MAFLD (all *P* < 0.05). Moreover, the severity of MAFLD was correlated with age, FT, LH, and SHBG. Multivariate regression analysis revealed that FSH was an independent risk factor for MAFLD as well as moderate-to-severe MAFLD. FSH showed an excellent diagnostic value, with an AUC value of 0.992. Age-adjusted FSH presents an AUC value of 0.996.

Univariate logistic regression analysis determined that LH and SHBG, but not TT affected the occurrence of MAFLD. Many studies have shown that serum testosterone concentrations decrease with age, which is partly due to a decrease in the number and function of interstitial cells in the testes and a decrease in the pulsatile secretion of LH that stimulates interstitial cells [37]. SHBG is a sex hormone-binding globulin synthesized by liver cells that protects sex hormones from adhesion as well as biological and chemical degradation [38]. The SHBG level in adult males gradually increases with age, leading to more testosterone binding to SHBG [38]. Several previous studies have demonstrated that a lower TT level is associated with a high prevalence of MAFLD [17–19], while other studies have found no significant association between the testosterone level and MAFLD [20, 21]. Moreover, in this study, multivariate regression analysis revealed that only FSH was an independent risk factor. Many previous studies only compared the parameters between the MAFLD and control groups but did not adjust for confounders [17–19], which may explain the controversial results.

In the current study, we found that FSH was an independent risk factor for MAFLD as well as moderate-to-severe MAFLD. FSH is a glycoprotein peptide hormone synthesized by the anterior pituitary gland that affects both the reproductive and nonreproductive systems. A recent study has demonstrated that FSH regulates hepatic lipid metabolism in mice [39]. Additionally, a cross-sectional study has reported that high FSH level is associated with MAFLD in men aged over 80 years old [40]. Moreover, a recent study in a Chinese elderly population (over 60 years old) consisting of both men and women has found that FSH is negatively associated with NAFLD in both men and women [41]. Our data were inconsistent with these reports, which may be due to the difference in study populations. The above studies enrolled an elderly population, while our study had a much younger population. Here, we also determined that FSH had an excellent diagnostic value for MAFLD, with an AUC of 0.992 alone and 0.996 after adjusting age confounder, indicating that FSH may be a potential diagnostic biomarker for MAFLD.

Our data suggest that MAFLD is related to male hormones. Previous studies have suggested a link between metabolic dysfunction and male reproductive health [42, 43]. Metabolic syndrome was reported to be associated with a decline in TT while no alterations in gonadotropin levels, and it was associated with hypogonadism, poor sperm morphology, testis ultrasound inhomogeneity, and erectile dysfunction [44]. A meta-analysis of 21 studies revealed a J-shaped association between body mass index and abnormal sperm count [45]. Obesity is negatively correlated with TT, LH, and SHBG [46], and affects male-factor infertility [47]. Our findings and these studies confirmed that metabolic dysfunction may affect male reproduction health by regulating male hormones. However, further experiments are needed to uncover the underlying mechanism.

This study has several limitations. First, this was a retrospective study and the inherent bias associated with such a study design cannot be avoided. Second, crucial data on marital or fertility status, and history of (in)fertility were not collected. Male sex hormones have been shown to correlate with male fertility status [48], which underscores the significance of these missing data. Therefore, our conclusion that FSH is a risk factor for MAFLD should be applied with caution due to the lack of male fertility data. Third, we did not collect data on semen parameters and testicular ultrasound. However, FSH plays a crucial role in the regulation of spermatogenesis [49]. and acts on Sertoli cells, the main component of the seminiferous tubules [50]. Increased levels of FSH may indicate abnormal spermatogenesis or testicular damage. Therefore, the findings of this study should be interpreted with careful consideration of these unexamined factors.

In conclusion, the present study identified that FSH is an independent risk factor for both MAFLD and moderate-to-severe MAFLD and that it had a diagnostic value for MAFLD, with an AUC value of 0.992 alone and 0.996 after adjusting the age confounder. Therefore, FSH may be a potential diagnostic and predictive biomarker for MAFLD, which warrants further clinical studies.

## Data Availability

All the data were included in this paper.
